# A Bisphosphonate With a Low Hydroxyapatite Binding Affinity Prevents Bone Loss in Mice After Ovariectomy and Reverses Rapidly With Treatment Cessation

**DOI:** 10.1002/jbm4.10476

**Published:** 2021-03-03

**Authors:** Abigail A Coffman, Jelena Basta‐Pljakic, Rosa M Guerra, Frank H Ebetino, Mark W Lundy, Robert J Majeska, Mitchell B Schaffler

**Affiliations:** ^1^ Department of Biomedical Engineering The City College of New York New York NY USA; ^2^ Department of Chemistry University of Rochester Rochester NY USA; ^3^ BioVinc, LLC Pasadena CA USA; ^4^ Department of Anatomy and Cell Biology Indiana University Indianapolis IN USA

**Keywords:** ANALYSIS/QUANTITATION OF BONE, ANTIRESORPTIVES, BONE HISTOMORPHOMETRY, BONE MICRO‐COMPUTED TOMOGRAPHY (μCT), BONE MODELING AND REMODELING, BONE QUANTITATIVE (QCT), DISEASES AND DISORDERS OF/RELATED TO BONE, OSTEOPOROSIS, THERAPEUTICS

## Abstract

Bisphosphonates (BPs) are a mainstay of osteoporosis treatment; however, concerns about bone health based on oversuppression of remodeling remain. Long‐term bone remodeling suppression adversely affects bone material properties with microdamage accumulation and reduced fracture toughness in animals and increases in matrix mineralization and atypical femur fractures in patients. Although a “drug holiday” from BPs to restore remodeling and improve bone quality seems reasonable, clinical BPs have long functional half‐lives because of their high hydroxyapatite (HAP) binding affinities. This places a practical limit on the reversibility and effectiveness of a drug holiday. BPs with low HAP affinity and strong osteoclast inhibition potentially offer an alternative approach; their antiresorptive effect should reverse rapidly when dosing is discontinued. This study tested this concept using NE‐58025, a BP with low HAP affinity and moderate osteoclast inhibition potential. Young adult female C57Bl/6 mice were ovariectomized (OVX) and treated with NE‐58025, risedronate, or PBS vehicle for 3 months to test effectiveness in preventing long‐term bone loss. Bone microarchitecture, histomorphometry, and whole‐bone mechanical properties were assessed. To test reversibility, OVX mice were similarly treated for 3 months, treatment was stopped, and bone was assessed up to 3 months post‐treatment. NE‐58025 and RIS inhibited long‐term OVX‐induced bone loss, but NE‐58025 antiresorptive effects were more pronounced. Withdrawing NE‐58025 treatment led to the rapid onset of trabecular resorption with a 200% increase in osteoclast surface and bone loss within 1 month. Cessation of risedronate treatment did not lead to increases in resorption indices or bone loss. These results show that NE‐58025 prevents OVX‐induced bone loss, and its effects reverse quickly following cessation treatment in vivo. Low‐HAP affinity BPs may have use as reversible, antiresorptive agents with a rapid on/off profile, which may be useful for maintaining bone health with long‐term BP treatment. © 2021 The Authors. *JBMR Plus* published by Wiley Periodicals LLC on behalf of American Society for Bone and Mineral Research.

## Introduction

Bisphosphonates (BPs) have been the standard antiresorptive therapy used to prevent bone loss in osteoporosis for nearly three decades.^(^ ^1^, ^2^, ^3^ ^)^ They act by inhibiting osteoclastic bone resorption, which suppresses remodeling thereby maintaining bone mass.^(^ ^4^, ^5^ ^)^ BPs are highly effective at reducing bone loss and preventing fractures in postmenopausal osteoporosis, men with osteoporosis, and patients receiving glucocorticoid treatments. Yet despite their efficacy in conserving bone mass and preventing osteoporotic fractures, long‐term suppression of bone remodeling can adversely affect bone tissue material properties. The consequences of long‐term BP use can include microdamage accumulation in bone,^(^ ^6^, ^7^, ^8^ ^)^ increases in matrix mineral content,^(^ ^9^, ^10^, ^11^ ^)^ and loss of tissue microstructural heterogeneity,^(^ ^12^, ^13^, ^14^ ^)^ all of which potentially contribute to decreased material‐level fracture resistance. Impaired bone quality after long‐term suppression of remodeling has been found in both animal models and tissues from patients treated for long periods with BPs,^(^ ^10^, ^11^, ^12^, ^14^, ^15^ ^)^ and is thought to underlie the atypical femur fractures (AFFs) observed in some patients after long‐term treatment with antiresorptives.^(^ ^16^, ^17^, ^18^, ^19^ ^)^ As reviewed by Shane et al,^(^ ^20^ ^)^ the absolute risk of AFFs in patients taking BPs is low, ranging from 3.2 to 50 cases per 100,000 person‐years. However, long‐term BP use is associated with a substantially higher risk of AFF (∼100 per 100,000 person‐years).

A temporary cessation of BP treatment, referred to as a “drug holiday,” has been widely discussed in the clinical literature as a means to restore bone remodeling and to recover bone material properties; however, its efficacy in restoring bone quality is unclear.^(^ ^21^, ^22^, ^23^, ^24^, ^25^, ^26^, ^27^ ^)^ A major challenge to implementing an effective drug holiday strategy is that the leading BPs in current clinical use (i.e., alendronate, risedronate (RIS), ibandronate, and zoledronate) have very long functional half‐lives in vivo, although some variation exists based on dose and specific compound. As a result, many BPs can remain in bone tissue for periods estimated to be years in humans.^(^ ^22^, ^26^, ^28^ ^)^

The ability to “target” or bind to bone mineral is one of the two principal criteria used to select BPs for development and clinical use. It has been assessed quantitatively by measuring binding affinity for hydroxyapatites (HAPs) in vitro, acute skeletal retention in vivo, or functional life in patients.^(^ ^29^, ^30^, ^31^, ^32^, ^33^ ^)^ Among the BPs in wide clinical use for preventing bone loss, zoledronate and alendronate bind to bone mineral with the highest affinity, followed by ibandronate and then RIS.^(^ ^29^, ^31^, ^34^ ^)^ The second criterion is the ability to inhibit osteoclast activity and its potency in this respect is measured by the ability to inhibit farnesyl pyrophosphate synthase (FPPS), an enzyme in the mevalonate pathway that is required for osteoclast ruffled border formation.^(^ ^35^, ^36^ ^)^ Among the widely used clinical BPs, zoledronate and RIS are more potent and thus prevent bone resorption at lower doses than ibandronate or alendronate.^(^ ^34^, ^36^ ^)^ Finally, other reported properties, including antiapoptotic activity,^(^ ^37^, ^38^, ^39^, ^40^ ^)^ safety,^(^ ^41^ ^)^ and skeletal distribution,^(^ ^4^, ^42^, ^43^ ^)^ may contribute to the clinical effects and usefulness of BPs.

**Fig 1:**
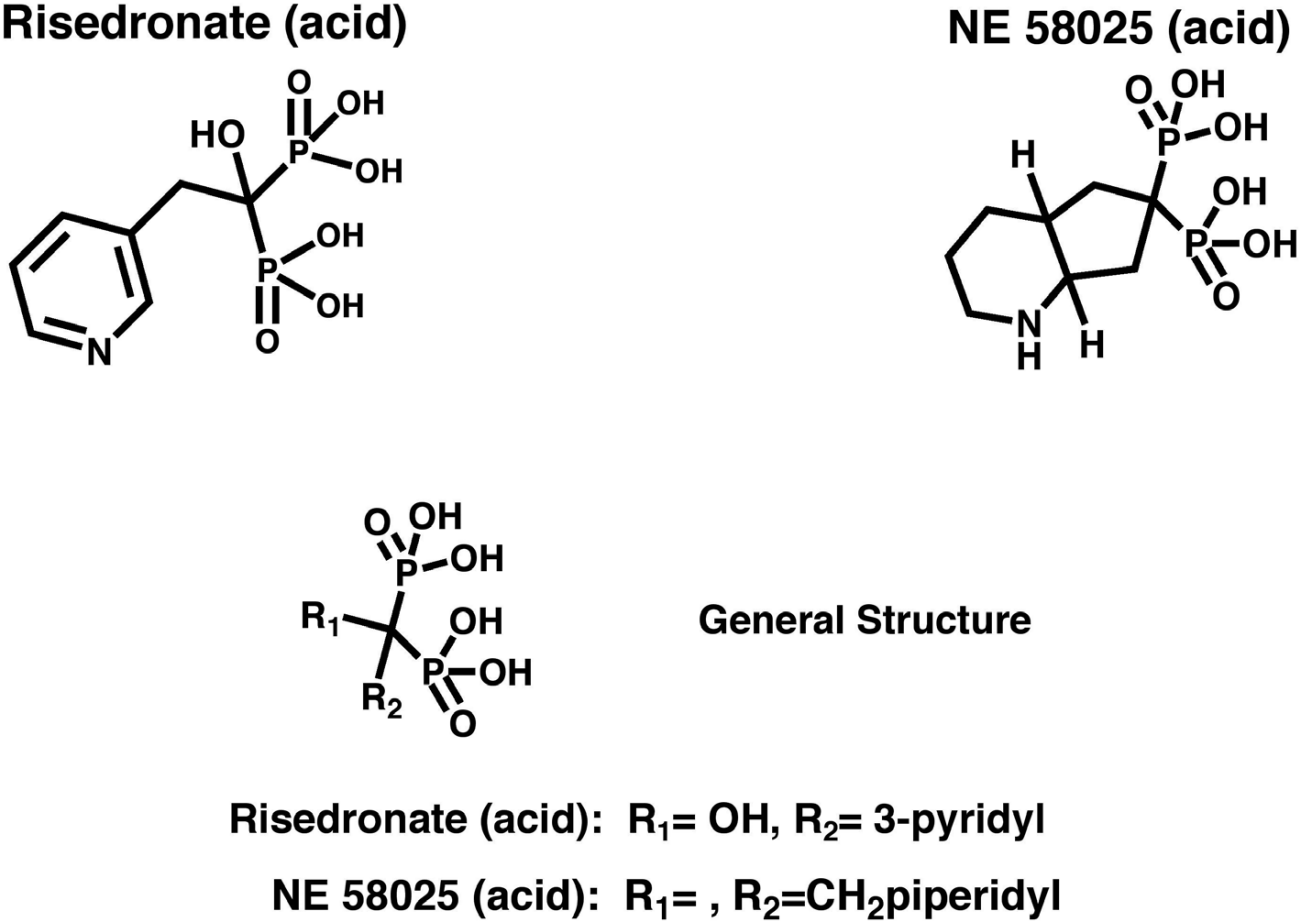
Chemical structure for risedronate and NE‐58025. The general structure for bisphosphonate has a phosphate–carbon–phosphate backbone, a R_1_ and a R_2_ group. R_1_, usually a hydroxyl group, is responsible for the affinity to hydroxyapatite; R_2_ accounts for the differences in potency.

Medicinal chemists have designed many variants of BPs over the years that have advanced the understanding of structural contributions to the pharmacological features of this drug class. These developments have ultimately identified highly efficacious BP drugs for development and clinical use. The key chemical feature of the BPs is the joining of two phosphonate moieties by one central carbon. This P‐C‐P structure was found to be metabolically stable versus the P‐O‐P feature of the natural bone metabolism regulator pyrophosphate; therefore, the BP chemical class provided useful drug candidates for systemic administration.^(^ ^34^, ^44^ ^)^ Both of these general chemical features uniquely coordinate to calcium and to the major calcium phosphate mineral component of bone—HAP. Variants can be compared and ranked for their bone affinity, using various in vitro models comprised of calcium phosphate HAP crystals and mineral surfaces.^(^ ^45^ ^)^ The BP structure also offers two additional binding sites to the central carbon, which are of course not available on the corresponding central divalent oxygen of pyrophosphate. These substituents are often referred to as R_1_ and R_2_ groups (Fig. 1), where one R group may be an additional calcium coordinating substituent, such as an –OH (hydroxyl) group that enhances BP bone affinity and is a characteristic substituent of almost all leading BPs in clinical use today.^(^ ^34^, ^46^, ^47^ ^)^ This extra coordinating group offers a more rapid and greater propensity for the distribution of a BP to bone surfaces because of tighter binding to those surfaces. A second R group substituent typically includes a nitrogen containing substituent, which if appropriately designed can offer an additional mineral binding site and perhaps even more importantly can yield BPs that bind tightly to a key enzyme target within osteoclasts (FPPS), leading to potent osteoclast inhibition and antiresorptive properties.^(^ ^3^, ^35^, ^47^ ^)^ Groups, such as pyridyl alkyl, imidazole alkyl, and amino alkyl, are typical substituents leading to structures such as RIS, zoledronate, and alendronate, respectively.

There is a subset of BPs that is comprised of potent FPPS inhibitors, which have low HAP binding affinity. These FPPS inhibitors should have the potential to prevent bone loss.^(^ ^30^, ^36^, ^48^ ^)^ However, their low HAP affinity could be hypothesized to allow these agents to clear more rapidly from the skeleton when dosing is stopped.^(^ ^33^ ^)^ In principle, this would allow bone remodeling to resume quickly and permit bone quality to recover; however, the use and reversibility of such potentially reversible BPs have not been well characterized in vivo. NE‐58025 (cis‐octahydro‐1‐pyrindine‐6, 6‐bisphosphonic acid; Fig. 1), a BP created by our coauthor (FHE) and initially reported in 1990 with good acute in vivo antiresorptive activity, meets these key criteria. This allows for an in vivo proof‐of‐concept test for a reversible BP with low HAP binding affinity and high FPPS inhibition.^(^ ^49^, ^50^ ^)^ NE‐58025 has a relatively high FPPS inhibitory potency, as do other clinically used BPs, such as zoledronate and RIS.^(^ ^36^, ^51^ ^)^ The R_1_ and R_2_ groups of NE‐58025 are incorporated into a cycloalkyl group that results in a lower bone affinity, likely by sterically limiting the binding of the phosphonate groups to calcium on HAP.^(^ ^49^, ^50^ ^)^ The low HAP binding affinity of NE‐58025 suggests that it should be more rapidly reversible than higher affinity, clinically used BPs once dosing stops, thus it is potentially valuable for exploring the on–off usefulness of such agents. Little is known about the efficacy of NE‐58025 in preventing long‐term bone loss; so far there are no in vivo data on its potential reversibility.

Here, we tested the effectiveness of low HAP binding affinity BPs (i.e., NE‐58025) in preventing long‐term bone loss after estrogen withdrawal in mice. Changes in trabecular bone, cortical bone, and whole‐bone mechanical properties were assessed. We then determined the time course of bone loss with NE‐58025 versus RIS and the extent of reversibility after stopping long‐term treatment with these two agents by assessing reactivation of resorption and bone loss.

## Materials and Methods

### Bisphosphonates

NE‐58025 (cis‐octahydro‐1‐pyrindine‐6, 6‐bisphosphonic acid), a BP with low HA affinity but with an osteoclast inhibition potency similar to RIS, was created by our coauthor (FHE) at Norwich Eaton Pharmaceuticals and initially reported in 1990.^(^ ^49^ ^)^ RIS was purchased from Sigma‐Aldrich. The rationale for choosing RIS was that NE‐58025 is reported in the literature as a general structural analogue with even lower binding affinity than RIS. In addition, human and rodent studies found that resorption indices show a more rapid rebound when RIS treatment is stopped than when other BPs (i.e., alendronate, zoledronate) treatments are suspended.^(^ ^22^, ^23^, ^52^, ^53^ ^)^ Thus, using RIS as our reference point let us gain insight into whether the rapid reversal effect we hypothesized for NE58025 would be better than that of clinical BPs in terms of reversibility.

### Mice

Young adult, female C57BL/6 mice (15 weeks old) were from the Jackson Laboratory. Mice underwent surgical ovariectomy (OVX) to induce estrogen deficiency, following procedures detailed elsewhere.^(^ ^54^ ^)^ OVX, sham‐OVX, baseline, and age‐matched controls were examined. All procedures were performed following Institutional Animal Care and Use Committee approval.

### Experiment 1. Short‐term prevention of bone loss by NE‐58025

In the first studies, we tested the efficacy of NE‐58025 in preventing bone loss after estrogen withdrawal. Following OVX, mice (N = 9‐10/group) were treated for 3 weeks with either PBS or NE‐58025 in PBS vehicle at pH 7.4. The least effective dose expected to prevent bone loss for NE‐58025 was previously reported to be in the range of 50–100 μg/kg of body weight.^(^ ^49^, ^50^ ^)^ For comparison, the dose of RIS used to prevent OVX‐induced bone loss in rodents has been reported in the range of 0.8–2.4 μg/kg.^(^ ^52^, ^55^ ^)^ NE‐58025 was administered by sc injection at 100 or 200 μg/kg daily; in addition, we examined the effect of intermittent treatment (100 μg/kg given 3 days/week; alternate days). Treatment continued for 3 weeks post‐OVX. Naïve 15‐week‐old mice were used as baseline controls and naïve aging controls (n = 5) were examined as well. During the study, mice had access to normal rodent chow and water *ad libitum*. Bone fluorochrome labels (calcein, 15 mg/kg ip; Sigma‐Aldrich) were given 9 and 2 days before the end of the studies. After euthanasia, the right femurs and tibia were fixed in 10% neutral buffered formalin and used for bone histomorphometry. Left femurs and tibia were frozen at −20°C for μCT studies as described below.

### Experiment 2. Long‐term suppression of bone loss and reversibility: Comparison of NE‐58025 and risedronate

In the second experiment, we (i) tested the long‐term efficacy of NE‐58025 in preventing bone loss after OVX based on the dose determined in Expt 1 and (B) assessed the time course and extent of reversibility after long‐term treatment with NE‐58025 versus RIS. For *the long‐term prevention study (Expt 2A)*, female B6 mice underwent OVX surgery as described in Expt 1. The BP treated mice received either NE‐58025 or RIS (n = 5/group). OVX/NE‐58025 mice received 200 μg/kg/day sc (PBS vehicle), the effective dose for preventing bone loss as determined in Expt 1. OVX/RIS mice were injected at 2.4 μg/kg sc (PBS vehicle), which has been reported to be 2–3 times the dose needed to prevent bone loss in ovariectomized rats.^52^, ^55^ ^)^ This RIS dose is the same as that used by Fuchs et al^(^ ^52^ ^)^ in their rat studies for BP reversibility. Mice were weighed weekly and per mouse BP doses adjusted if needed. The remaining group of OVX mice (n = 5) were injected with PBS vehicle. Age‐matched controls and sham‐operated mice were examined as well (n = 5/group). For these long‐term bone loss prevention studies, mice were treated for 3 months and then euthanized for μCT, biomechanics, and histomorphometric analyses. For the bisphosphonate withdrawal ‐ reversibility studies (Expt 2B), NE‐58025 or RIS treatment was carried out in OVX mice for 3 months, as above. Treatment was then stopped and groups of mice (n = 5) were then sacrificed each month up to 3 months posttreatment. Mice were weighed weekly and BP doses adjusted as needed. Throughout the studies, mice had access to normal rodent chow and water *ad libitum*. Bone fluorochrome labels (calcein, 15 mg/kg ip) were given 9 and 2 days before sacrifice. Femurs and tibia were harvested and processed as in the short‐term study discussed above.

### μCT imaging

MicroCT studies were performed to assess bone microarchitecture. Femurs were scanned using the SkyScan 1172 system (Bruker microCT) at a nominal isotropic voxel resolution of 6.7 μm. Images were acquired using a 10‐MP detector, 10‐W power energy setting (100 KV and 100 mA), and a 0.5‐mm aluminum filter to minimize beam‐hardening effects by filtering low‐energy photons. An alignment procedure and flat‐field detector calibration were performed before each scan to minimize ring artifacts and increase signal‐to‐noise ratio. Imaging, filtering and thresholding were performed following previous studies.^(^ ^56^, ^57^, ^58^ ^)^ HAP phantoms of known densities (0.25 and 0.75 g/cm^3^) and cross‐sectional dimensions similar to that of a mouse femur (2 mm diameter) were included in each scan. 3D reconstructions were obtained using a customized back‐projection algorithm (NRecon v1.6; Bruker, Belgium) with the following reconstruction parameters: post‐alignment compensation to optimize the center of rotation, smoothing correction kernel size of 1 pixel for asymmetrical boxcar window, ring artifact correction set to 10, beam hardening correction (40%). All settings were kept constant throughout each scan reconstruction. The μCT gray‐scale data set was transformed into Hounsfield units (HUs), defining a HU value of 0 for saline and a HU value of −1000 to black pixels (air). The linear relationship between the HU and the HAP calibration phantoms was used to determine voxel tissue‐mineral densities. Global thresholding was performed by setting a bone‐equivalent threshold to 0.45 g/cm^3^ for all scans as per Palacio‐Mancheno et al.^(^ ^56^ ^)^

CT Analyzer software (version 1.13; Bruker) was used for measuring cortical and trabecular microarchitecture. Analyses were performed by one of two observers (AC, JB‐P) who were blinded to specimen identity. For trabecular bone, trabecular bone volume fraction (BV/TV), trabecular thickness (Tb.Th), and trabecular number (Tb.N) were measured in the metaphysis at a set distance from the distal process of the metaphyseal growth plate. Trabecular bone volume of interest (VOI) was defined as 10% of the bone length. In long‐term bone‐loss prevention studies (mouse aged 4–7 months), we observed increases in femur lengths for all groups compared with baseline. The small amount of elongation of femurs in control groups (400 ± 150 μm vs baseline) is not unexpected given that the growth plate at the distal femur remained open at the start of these studies. We observed a greater amount of elongation in OVX mice with femurs increasing in length by 750 ± 140 μm vs baseline. This is consistent with Wronski et al who reported that OVX and estrogen withdrawal leads to elongation of long bones in young adult rodents because estrogen loss delays growth plate closure.^(^ ^59^, ^60^ ^)^ Increases in bone length over the course of the study were unaffected by BP treatment. Finally, there was no consistent increase in bone length during the 3‐month period after stopping BP treatment (mouse aged 7–10 months). Antiresorptives, like BPs, are known to prevent the developmentally programmed resorption and reduction in trabecular number that normally occurs just below the growth plate, which results in an increase in bone volume that does not reflect anabolic activity.^(^ ^61^, ^62^ ^)^ To compensate for the increase in bone length and to avoid artifactually elevated measurements of bone volume in OVX and control mice in these long‐term studies, we shifted the trabecular bone μCT measurement region away from the growth plate by the average elongation distance for each group (750 and 400 μm for OVX and controls, respectively). This correction assumed that most of the bone elongation occurred at the distal femoral growth plate in these mice, which was reasonable given the femoral head growth plate in mice starts to fuse by approximately 4 months of age.^(^ ^63^ ^)^ Cortical bone was analyzed at the mid‐diaphyseal region (mid‐diaphysis ±5% of bone length), and total cross‐sectional area (Tt.Ar), cortical bone area (Ct.Ar), cortical area fraction (Ct.Ar/Tt.Ar), and polar of inertia (J) were measured.

### Bone histomorphometry

Right femurs and tibias were fixed in 10% neutral buffered formalin, dehydrated, and embedded in methyl methacrylate. For trabecular bone, longitudinal 5–7‐μm thick sections were cut from the metaphyseal regions using a Leica Supercut microtome equipped with a tungsten carbide knife. Eroded surface/bone surface (ES/BS; %), osteoclast surface/bone surface (Oc.S/BS; %), osteoblast surface/bone surface (Ob.S/BS; %), and osteoid surface/bone surface (OS/BS; %) were measured on toluidine blue‐stained sections. For cortical bone, cross sections were cut at a thickness of 200 μm from the mid‐diaphysis using a diamond wafering saw (Leica SP 1600) and polished to 90 μm. Bone fluorochrome labeling (mineral appositional rate [MAR]; μm/day) and mineralizing surface/bone surface [MS/BS]; %) were measured from unstained sections for both cancellous and cortical bone. Histomorphometry measurements were made using an OsteoMeasure system and were made by a single observer who was blinded to section identity.

### Biomechanical testing

Four‐point bending to failure was used to test whole‐bone mechanical properties. Left femurs were thawed and equilibrated in PBS at room temperature for 2 hours before undergoing testing. Intact bones were positioned in the test apparatus with the posterior side in compression and anterior side in tension. Bending tests were conducted in the posterior‐to‐anterior direction with a rate of 0.05 mm/s using the Bose ElectroForce 3200 system (Bose Corp) following procedures previously reported from our laboratory.^(^ ^64^, ^65^, ^66^ ^)^ Span lengths for inner‐ and outer‐contact points were 2.2 and 6.35 mm, respectively. Whole‐bone mechanical properties, including stiffness, yield point (defined using a 10% offset method), ultimate load, energy to fracture, and postyield displacement, were determined from load‐displacement curves using a custom‐written software code in MatLab (MathWorks).

### Statistical analyses

Comparisons were preformed using Kruskal‐Wallis nonparametric test with Dunn's post hoc test. In experiments 1 and 2A, the primary comparisons were made against baseline controlto test for drug efficacy in preventing bone loss. Differences in the BP withdrawal studies (experiment 2B) were assessed relative to the respective 3‐month treatment groups (OVX/NE‐58025 or OVX/RIS), which were defined as the starting points for the BP reversal studies. GraphPad Prism software (GraphPad) was used for all statistical analyses. Data are reported as mean ± SD.

## Results

Mice tolerated all procedures well and there were no significant differences in body weights noted among BP and age‐matched groups during the short‐term or long‐term BP studies. As expected, body weights increased approximately 25% in OVX mice during the long‐term studies. These increases were similar in BP and PBS vehicle‐treated mice (*p* = 0.001; Table 1).

**Table 1:** Mouse Body Weights at Baseline and 3 Months After Ovariectomy

Parameter	Baseline	OVX/PBS	OVX/NE‐025	OVX/RIS	Age‐matched control (7 mo)
Weight, g	22.4 ± 1.3	29.1 ± 1.4^*^	27.8 ± 1.3^*^	28.4 ± 1.8^*^	24.6 ± 2.5^*^

Treatments with vehicle (OVX/PBS), NE‐58025 (OVX/NE‐025), or risedronate (OVX/RIS) treatment. Data shown as mean ± SD.

Abbreviations: OVX = Ovariectomy; RIS = risedronate.

*p = 0.001 for OVX/PBS, OVX/NE‐025, and OVX/RIS vs baseline control; *p* = 0.002 for age‐matched control vs baseline.

### Experiment 1: Short‐term prevention of bone loss after OVX with NE‐58025

**Fig 2:**
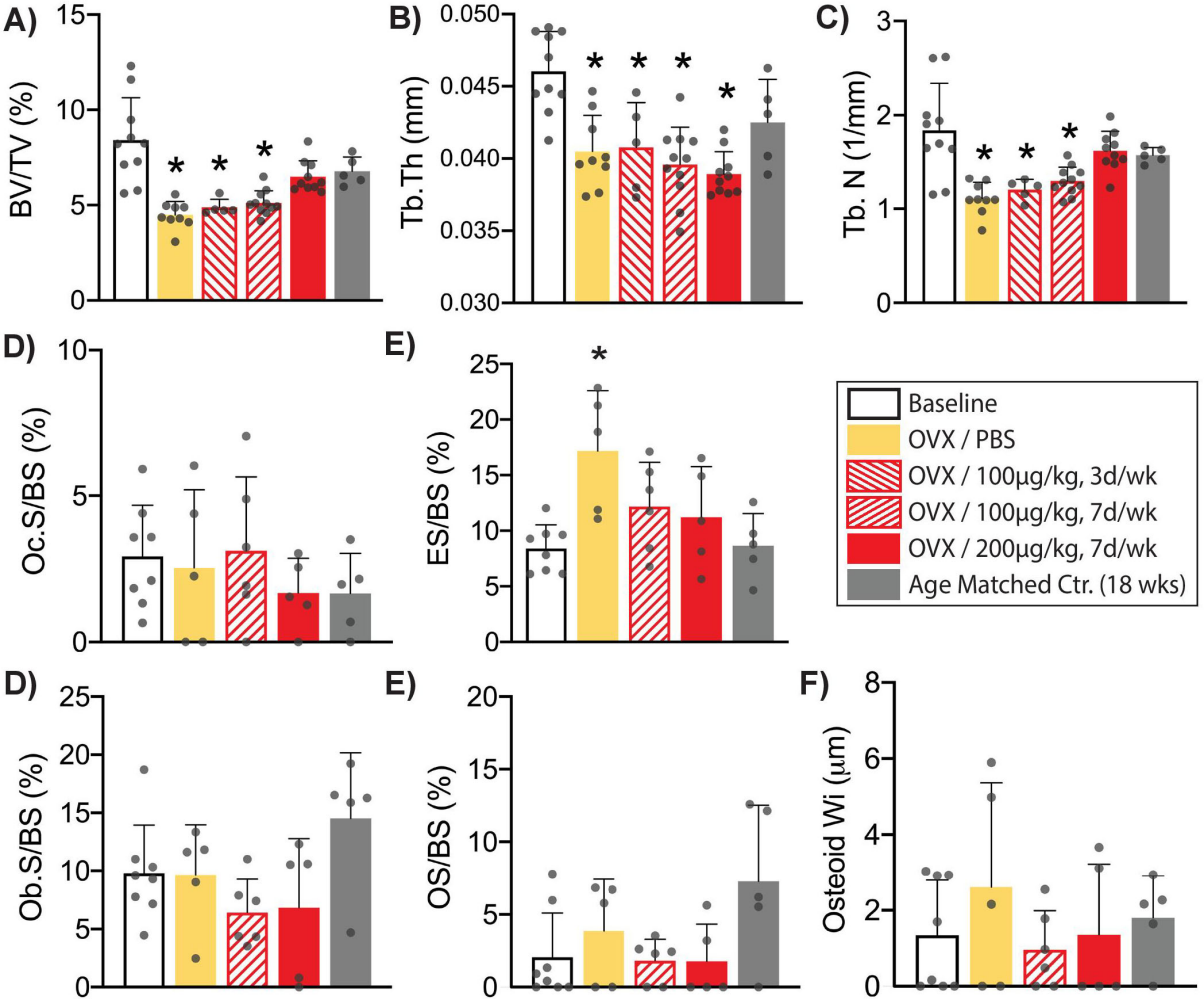
Changes in cancellous bone volume and bone cell surface measurements for short‐term NE‐58025 studies to determine effective dose for preventing OVX‐induced bone loss. Graphs *A*‐*C* show architectural changes from μCT analyses. All comparisons expressed versus baseline controls. (*A*) Bone volume fraction (BV/TV) decreased significantly after ovariectomy (OVX) in OVX/PBS, OVX/100 μg/kg, 3 days/week and OVX/100 μg/kg, 7 days/week (*p = 0.0001 for OVX/PBS and *p* = 0.0003 for OVX/100 μg/kg and OVX/100 μg/kg, 7 days/week). NE‐58025 given at 200 μg/kg, 7 days/week prevented OVX‐induced bone loss (*p* = 0.24). (*B*) Trabecular thickness (Tb.Th) decreased in all OVX groups (*p = 0.003 for OVX/PBS, *p* = 0.01 for OVX/100 μg/kg 3 days/week and *p* = 0.001 for OVX/100 μg/kg and OVX/200 μg/kg 7 days/week. (*C*) Trabecular number (Tb.N) was reduced for OVX/PBS, OVX/100 μg/kg 3 days/week and OVX/100 μg/kg 7 days/week (*p = 0.0001, *p* = 0.004, and *p* = 0.003, respectively). (*D*) Osteoclast surface/bone surface (Oc.S/BS) did not differ among all groups. (*E*) Eroded surface/bone surface (ES/BS) increased within 3 weeks post‐OVX in vehicle‐treated mice (*p = 0.004). There were no differences among groups in (*F*) osteoblast surface/bone surface (Ob.S/BS), (*G*) osteoid surface/bone surface (OS/BS), or (*H*) osteoid width (Osteoid Wi). Data shown as mean ± SD.

OVX resulted in significant trabecular bone loss (45%) over the 3‐week period versus control (*p* = 0.0001; Fig. 2*A*). NE‐58025 treatment at 100 μg/kg for 3 or 7 times per week attenuated but did not prevent bone loss after OVX (*p* = 0.0003 vs baseline control). In contrast, NE‐58025 given daily at 200 μg/kg successfully prevented this bone loss; BV/TV was not significantly different from baseline control (*p* = 0.24), and there was no increase in eroded surface versus baseline controls (Fig. 2*D*,*E*). Bone formation parameters did not differ for OVX/NE‐58025 given daily at 200 μg/kg versus baseline control bones (Table 2). BP treatment has no effect on bone in shams; these data are summarized in Supplemental Table S1. Based on these results, the 200 μg/kg/day dose of NE‐58025 was used for the long‐term prevention and reversal studies.

**Table 2:** Cancellous Bone‐Labeled Surface Measurements After 3 Weeks of NE‐58025 (NE‐025) Treatment Including MS/BS and MAR

Parameter	Baseline	OVX/PBS	OVX/NE‐025 100 μg/kg	OVX/NE‐025 200 μg/kg	Age‐matched control (18 wk)
MS/BS, %	11.0 ± 0.9	33.8 ± 6.9^*^	27.8 ± 9.6^*^	22.2 ± 1.9	32.9 ± 3.5^*^
MAR, μm/d	0.52 ± 0.1	0.78 ± 0.2	0.55 ± 0.1	0.94 ± 0.4	0.97 ± 0.4

Comparisons vs baseline control. Data shown as mean ± SD.

Abbreviations: MAR = mineral apposition rate; MS/BS = mineralizing surface/bone surface; OVX = ovariectomy.

*p = 0.004 for OVX/PBS, *p* = 0.02 for OVX/NE‐025, and *p* = 0.001 for age‐matched control.

### Experiment 2A: Long‐term prevention of bone loss after OVX using NE‐58025 or RIS

#### Trabecular bone

**Fig 3:**
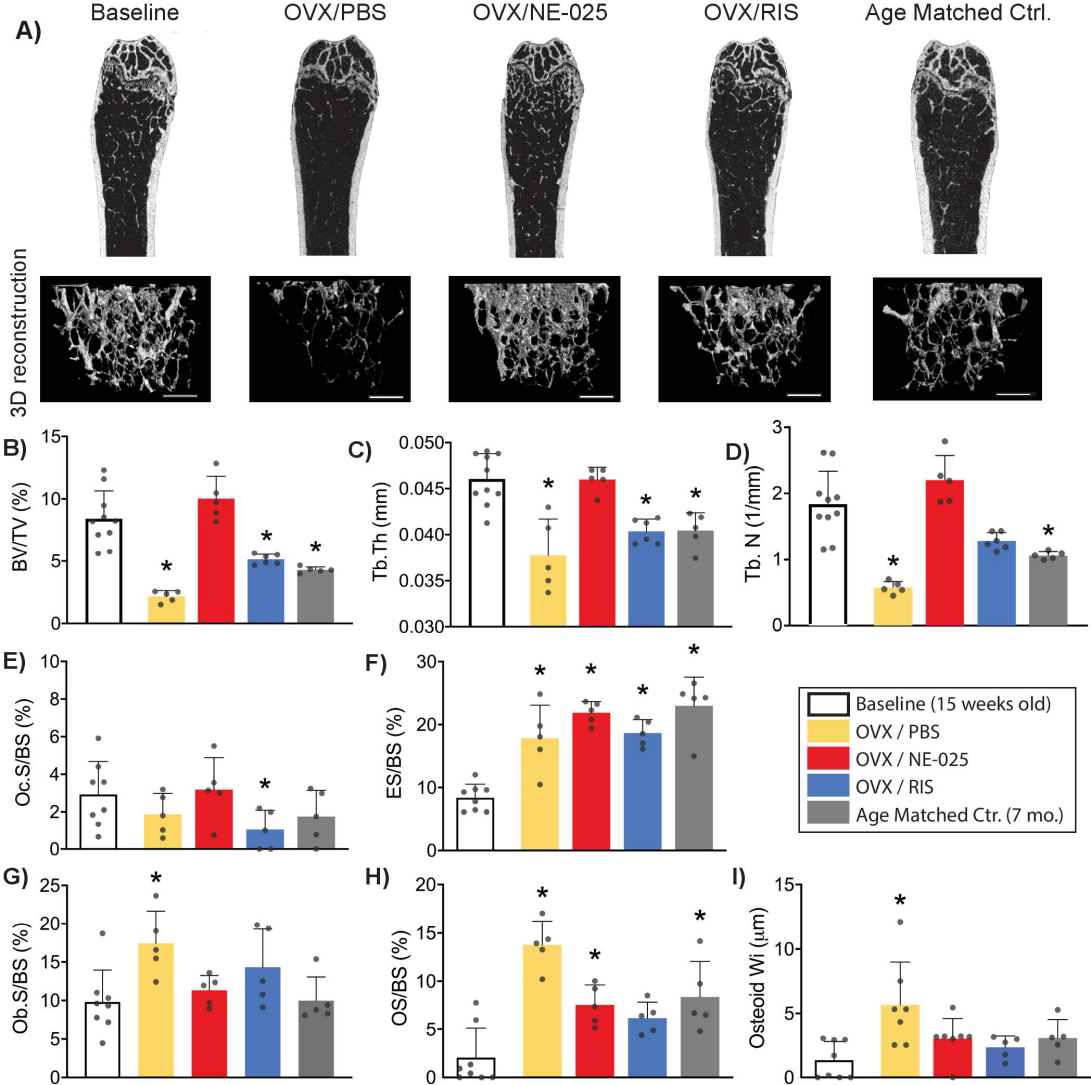
Changes in cancellous bone at 3 months post‐OVX with NE‐58025 (NE‐025) or RIS treatment. (*A*) Longitudinal and three‐dimensional μCT images of distal femurs for baseline, 3‐month treatment groups, and age‐matched controls (7 months old). Scale bar = 0.5 mm. Graphs *B*‐*D* show architectural changes from μCT analyses. All comparisons versus baseline control. (*B*) Decrease in trabecular bone volume fraction caused by OVX was prevented with NE‐025 (*p* = 0.5) but not with risedronate treatment (*p = 0.0001 for OVX/PBS, *p* = 0.048 for OVX/RIS, *p* = 0.003 for age‐matched control). (*C*) Trabecular thickness decreased as a result of OVX (*p = 0.001 for OVX/PBS) and aging (*p = 0.009 for age‐matched control). Tb.Th did not decline with NE‐025 treatment and slightly decreased with RIS (*p = 0.003). (*D*) Trabecular number decreased by 70% in OVX/PBS mice (*p = 0.0001) and ~40% for age matched controls (*p = 0.006). Graphs E‐I show changes in resorption and formation indices. (*E*) Osteoclast surface was decreased slightly for OVX/RIS (*p = 0.04). (*F*) Eroded surface increased for all groups (*p = 0.02 for OVX/PBS, *p* = 0.001 for OVX/NE‐025, *p* = 0.02 for OVX/RIS and *p* = 0.0001 for age matched controls). For OVX/PBS mice, formation indices increased significantly. (*G*) Osteoblast surface (*p = 0.002). (*H*) Osteoid surface (*p = 0.0001 for OVX/PBS, *p* = 0.04 for OVX/NE‐025 and *p* = 0.03 for age‐matched controls). (*I*) Osteoid width (*p = 0.002). Data shown as mean ± SD. BV/TV = trabecular bone volume fraction; ES/BS = eroded surface/bone surface; Ob.S/BS = osteoblast surface/bone surface; Oc.S/BS = osteoclast surface/bone surface; OS/BS = osteoid surface/bone surface; Osteoid Wi = osteoid width; OVX = ovariectomy; RIS = risedronate; Tb.Th = trabecular thickness; Tb.N = trabecular number.

**Fig 4:**
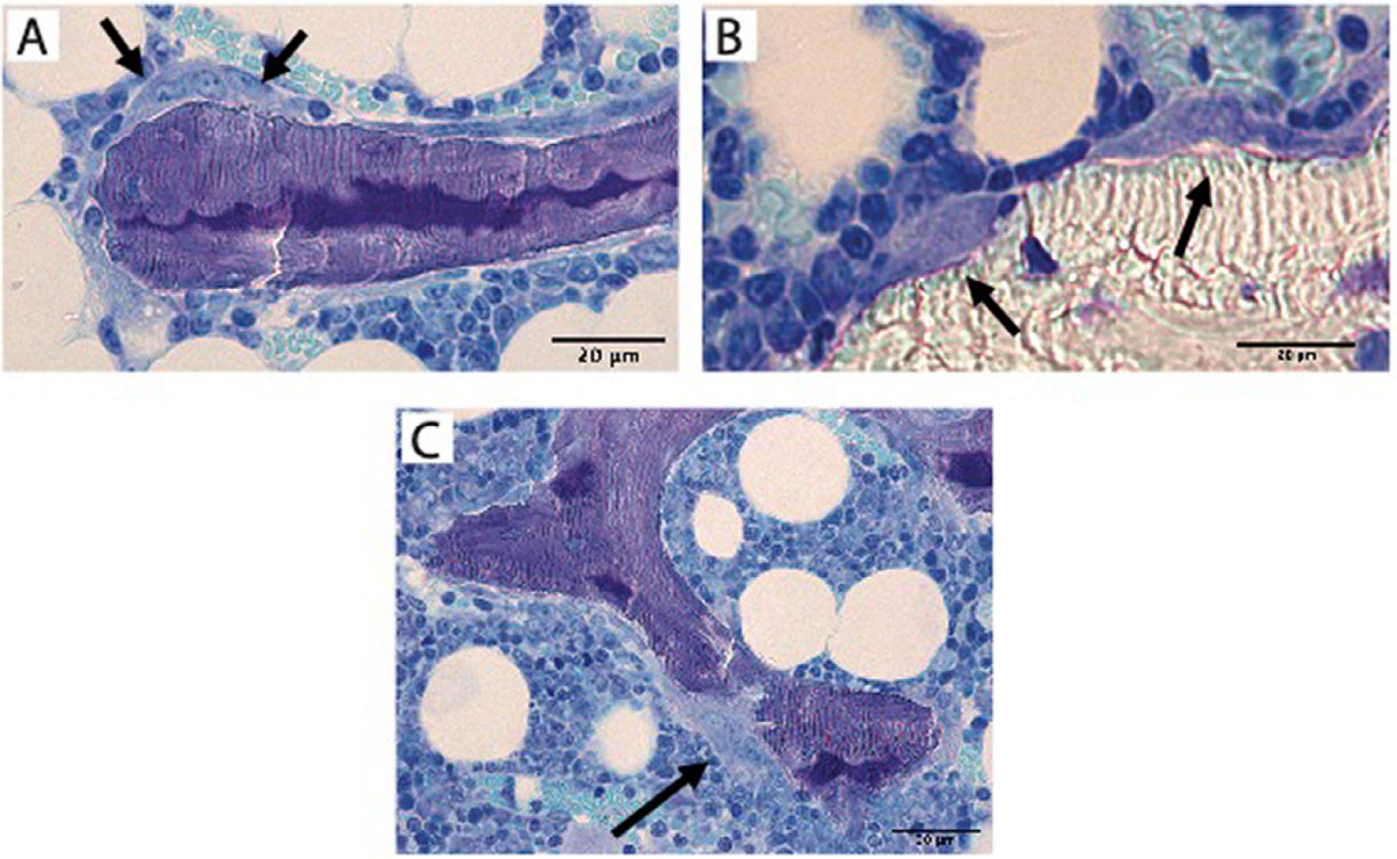
Toluidine blue‐stained bone sections of cancellous bone. (*A*) Shows a large osteoclast (arrows) on bone surface from long‐term OVX/NE‐025 treated bone; the cell is not located within a Howship's lacuna. (*B*) Shows several osteoclasts within Howship's lacunae at 1 month after stopping treatment (OVX/NE‐ 025 + 1‐month reversal). (*C*) Shows extensive eroded surface and a large osteoclast (arrow) that appears close to perforating through the trabecular element at 2 months after treatment cessation (OVX/NE‐025 + 2‐month reversal). Scale bar = 20 μm.

OVX resulted in an approximately 80% reduction in BV/TV versus baseline control (Fig. 3*B*) in the 3 months following estrogen withdrawal with corresponding decreases in trabecular thickness (−20%; Fig. 3*C*) and trabecular number (−65%; Fig. 3*D*). Bone loss did not occur in OVX mice treated with NE‐58025. Long‐term RIS treatment reduced OVX‐induced losses of trabecular bone by ~40%, but bone volume in this group was still reduced from baseline control (*p* = 0.05). Three months after OVX, osteoclast surface was similar among all groups (Fig. 3*E*). Osteoclasts were present on bone surfaces for both NE‐58025 and RIS treatment, consistent with the fact that BPs do not prevent osteoclasts from forming but markedly attenuate their ability to resorb bone. Eroded surface was increased in all groups compared with baseline animals (Fig. 3*F*). NE‐58025 and RIS groups exhibited very shallow resorption spaces (Fig. 4). Mineralizing surface increased with OVX and BP treatment (Table 3A).

**Table 3:** Cancellous Bone‐Formation Indices at (A) 3 Months Post‐OVX With NE‐58025 (NE‐025) or RIS Treatment (Comparisons vs Baseline Control: *p = 0.003 for OVX/PBS, *p* = 0.007 for OVX/NE‐025 and *p* = 0.001 for OVX/RIS), and (B) up to 3 Months After Stopping Bisphosphonate Treatment

(A)					
Parameter	Baseline	OVX/PBS	OVX/NE‐025	OVX/RIS	Age‐matched control (7 mo)
MS/BS, %	11.0 ± 0.9	32.7 ± 4.1^*^	24.2 ± 3.7^*^	35.2 ± 4.6^*^	22.4 ± 5.2
MAR, μm/d	0.52 ± 0.1	0.81 ± 0.2	1.15 ± 0.4^*^	1.6 ± 0.5^*^	0.97 ± 0.3

(B)							
Parameter	OVX/NE‐025,+1 mo	OVX/NE‐025,+2 mo	OVX/NE‐025,+3 mo	OVX/RIS,+1 mo	OVX/RIS,+2 mo	OVX/RIS,+3 mo	Age‐matched control (10 mo)
MS/BS, %	13.7 ± 5.7^***^	21.5 ± 7.2	25.2 ± 4.9	12.0 ± 9.3^**^	18.6 ± 8.9^**^	32.9 ± 5.6	35.0 ± 5.1
MAR, μm/d	0.78 ± 0.38	0.81 ± 0.24	0.98 ± 0.1	0.92 ± 1.24	1.75 ± 0.91	1.01 ± 0.2	0.73 ± 0.5

Data shown as mean ± SD.

Abbreviations: MAR = mineral apposition rate; MS/BS = mineralizing surface/bone surface; OVX = ovariectomy; RIS = risedronate.

Comparisons vs 3 mo of RIS treatment (OVX/RIS): ^**^ *p* = 0.002 for OVX/RIS, +1 mo, *p* = 0.01 for OVX/RIS, +2 mo. ^***^ *p* = 0.03 for OVX/NE‐025 + 1 mo vs 3 mo of NE‐58025 treatment.

#### Cortical bone

Long‐term OVX caused endocortical bone loss with an approximately 10% decrease in cortical area fraction versus baseline control caused by marrow cavity expansion. There were no significant changes in total bone area or moment of inertia at midfemoral diaphysis (Table 4A). Treatment with both NE‐58025 and RIS prevented marrow cavity expansion after OVX. There were no significant differences in mineralizing surface or mineral apposition (Table 5A).

**Table 4:** Cortical Bone Architecture: (A) At 3 Months Post‐OVX of NE‐58025 (NE‐025) or RIS Treatment, and (B) up to 3 Months After Stopping Bisphosphonate Treatment

(A)					
Parameter	Baseline	OVX/PBS	OVX/NE‐025	OVX/RIS	Age‐matched control (7 mo)
Ct.Ar/Tt.Ar, %	45.19 ± 1.9	41.61 ± 0.93^*^	45.47 ± 1.16	43.57 ± 1.79	46.93 ± 0.93
Tt.Ar, mm^2^	1.71 ± 0.15	1.78 ± 0.07	1.74 ± 0.06	1.76 ± 0.08	1.71 ± 0.98
Ct.Ar, mm^2^	0.77 ± 0.05	0.74 ± 0.04	0.79 ± 0.01	0.77 ± 0.04	0.80 ± 0.05
Ma.Ar, mm^2^	0.94 ± 0.1	1.04 ± 0.03^*^	0.95 ± 0.05	0.99 ± 0.06	0.91 ± 0.05
J, mm^4^	0.35 ± 0.05	0.34 ± 0.03	0.36 ± 0.02	0.35 ± 0.03	0.36 ± 0.04

(B)							
Parameter	OVX/NE‐025, +1 mo	OVX/NE‐025,+2 mo	OVX/NE‐025,+3 mo	OVX/RIS,+1 mo	OVX/RIS,+2 mo	OVX/RIS, +3 mo	Age‐matched control (10 mo)
Ct.Ar/Tt.Ar, %	43.92 ± 0.52	40.61 ± 1.99^**^	45.13 ± 0.81	43.28 ± 2.15	39.54 ± 1.1^***^	40.44 ± 1.77^***^	45.87 ± 1.52
Tt.Ar, mm^2^	1.74 ± 0.07	1.83 ± 0.1	1.9 ± 0.05^**^	1.82 ± 0.08	1.91 ± 0.11^***^	1.98 ± 0.11^***^	1.8 ± 0.09
Ct.Ar, mm^2^	0.76 ± 0.03	0.74 ± 0.02	0.86 ± 0.03	0.79 ± 0.06	0.75 ± 0.04	0.8 ± 0.01	0.83 ± 0.02
Ma.Ar, mm^2^	0.98 ± 0.05	1.09 ± 0.09^**^	1.05 ± 0.06	1.03 ± 0.05	1.15 ± 0.08^***^	1.18 ± 0.1^***^	0.98 ± 0.07
J, mm^4^	0.34 ± 0.02	0.36 ± 0.03	0.42 ± 0.02^**^	0.38 ± 0.04	0.38 ± 0.04	0.42 ± 0.03^***^	0.38 ± 0.03

Data shown as mean ± SD. (A) Ovariectomy resulted in almost a 10% decrease in Ct.Ar/Tt.Ar after 3 mo (*p = 0.004 vs baseline control) and an increase in Ma.Ar (*p = 0.02 vs baseline control), whereas bisphosphonate treatment prevented this bone loss. (B) NE‐58025 mice experienced increases in Tt.Ar and J after 3 mo of stopping treatment (**p = 0.01 for Tt.Ar, *p* = 0.02 for J vs OVX/NE‐025). Two mo after stopping RIS treatment there were reductions in Ct.Ar/Tt.Ar (^***^ *p* = 0.005 for OVX/RIS + 2 mo, *p* = 0.03 for OVX/RIS + 3 mo vs OVX/RIS) and Tt.Ar (^***^ *p* = 0.02 for OVX/RIS + 2 mo, *p* = 0.003 for OVX/RIS + 3 mo vs OVX/RIS). J increased by 3 mo after stopping RIS treatment (^***^ *p* = 0.007 vs OVX/RIS).

Abbreviations: Ct.Ar = Cortical area fraction; J = polar moment of inertia; Ma.Ar = marrow area; OVX = ovariectomy; RIS = risedronate; Tt.Ar = total cross‐sectional area.

**Table 5:** Resorption Activity and Labeled Bone Surfaces of Cortical Bone: (A) After 3 Months of NE‐58025 or RIS Treatment, and (B) After Stopping Bisphosphonate Treatment

(A)						
Parameter	Baseline	OVX/PBS	OVX/NE‐025	OVX/RIS	Age‐matched control (7 mo)	Sham (7 mo)
Ec. ES/BS, %	2.73 ± 2.1	12.96 ± 2.8^*^	3.19 ± 4.1	0.60 ± 0.9	4.17 ± 4.0	2.22 ± 4.2
Ec. MS/BS, %	7.05 ± 4.3	5.64 ± 2.4	1.0 ± 2.2^**^	3.66 ± 4.5	18.03 ± 5.8	1.0 ± 1.6
Ec. MAR, μm/d	0	0	0	0.17 ± 0.4	0.07 ± 0.1	0

(B)					
Parameter	OVX/NE‐025,+1 mo	OVX/NE‐025,+2 mo	OVX/RIS,+1 mo	OVX/RIS,+2 mo	Age matched ctrl (10 mo)
Ec. ES/BS, %	2.68 ± 0.8	2.95 ± 2.8	3.70 ± 2.1	3.95 ± 6.0	2.63 ± 2.5
Ec. MS/BS, %	6.36 ± 3.1	15.53 ± 7.4	4.10 ± 4.9	7.34 ± 7.7	19.3 ± 9.7
Ec. MAR, μm/d	0	1.12 ± 1.2^**^	0	1.76 ± 2.5	1.81 ± 1.3

Data presented as mean ± SD. (A) Ovariectomy resulted in an increase in ES/BS (*p = 0.04 vs baseline control). ** *p* = 0.02 for OVX/NE‐025 for Ec. MS/BS. (B) Data for NE‐58025 mice showed an increase in MAR after 2 mo (**p = 0.003 vs OVX/NE‐025).

Abbreviations: Ec. ES/BS = Endocortical eroded surface; Ec. MAR = endocortical mineral apposition rate; Ec. MS/BS = endocortical mineralizing surface/bone surface; OVX = ovariectomy; RIS = risedronate.

#### Mechanical properties

Whole‐bone strength, stiffness, and work to fracture were not changed significantly by OVX (*p* = 0.4; Table 6A) or by treatment with either NE‐58025 or RIS (*p* = 0.45 and 0.5, respectively; Table 6A) compared with baseline control. This finding was consistent with no changes in total cortical bone area or moment of inertia noted in μCT studies.

**Table 6:** Biomechanical Data from Femoral Bending Tests: (A) at 3 Months Post‐OVX With NE‐58025 (NE‐025) or RIS Treatment and (B) After Stopping Bisphosphonate Treatment

(A)					
Parameter	Baseline	OVX/PBS	OVX/NE‐025	OVX/RIS	Age matched control (7 mo)
Stiffness, N/mm	103.9 ± 12.2	105.9 ± 32.9	130.1 ± 41.2	118.7 ± 29.4	140.1 ± 31.2
Ultimate force, N	28.4 ± 4.3	25.7 ± 1.4	29.73 ± 4.0	25.35 ± 1.4	29.5 ± 2.2
Postyield displacement, mm	0.25 ± 0.1	0.22 ± 0.01	0.21 ± 0.05	0.17 ± 0.04	0.17 ± 0.05

(B)					
Parameter	OVX/NE‐025,+1 mo	OVX/NE‐025,+2 mo	OVX/RIS,+1 mo	OVX/RIS,+2 mo	Age Matched Control (10 mo)
Stiffness, N/mm	104.7 ± 27	149.7 ± 27.3	110.8 ± 30.9	121.2 ± 35.5	102.1 ± 17.8
Ultimate force, N	24.66 ± 2.08	28.85 ± 2.58	22.87 ± 4.1	26.04 ± 2.25	29.98 ± 4.42
Postyield displacement, mm	0.19 ± 0.05	0.22 ± 0.08	0.18 ± 0.07	0.20 ± 0.07	0.14 ± 0.08

Data shown as mean ± SD.

Abbreviations: OVX = Ovariectomy; RIS = risedronate.

### Experiment 2B: Effect of NE‐025 and RIS withdrawal after long‐term treatment

#### Trabecular bone

**Fig 5:**
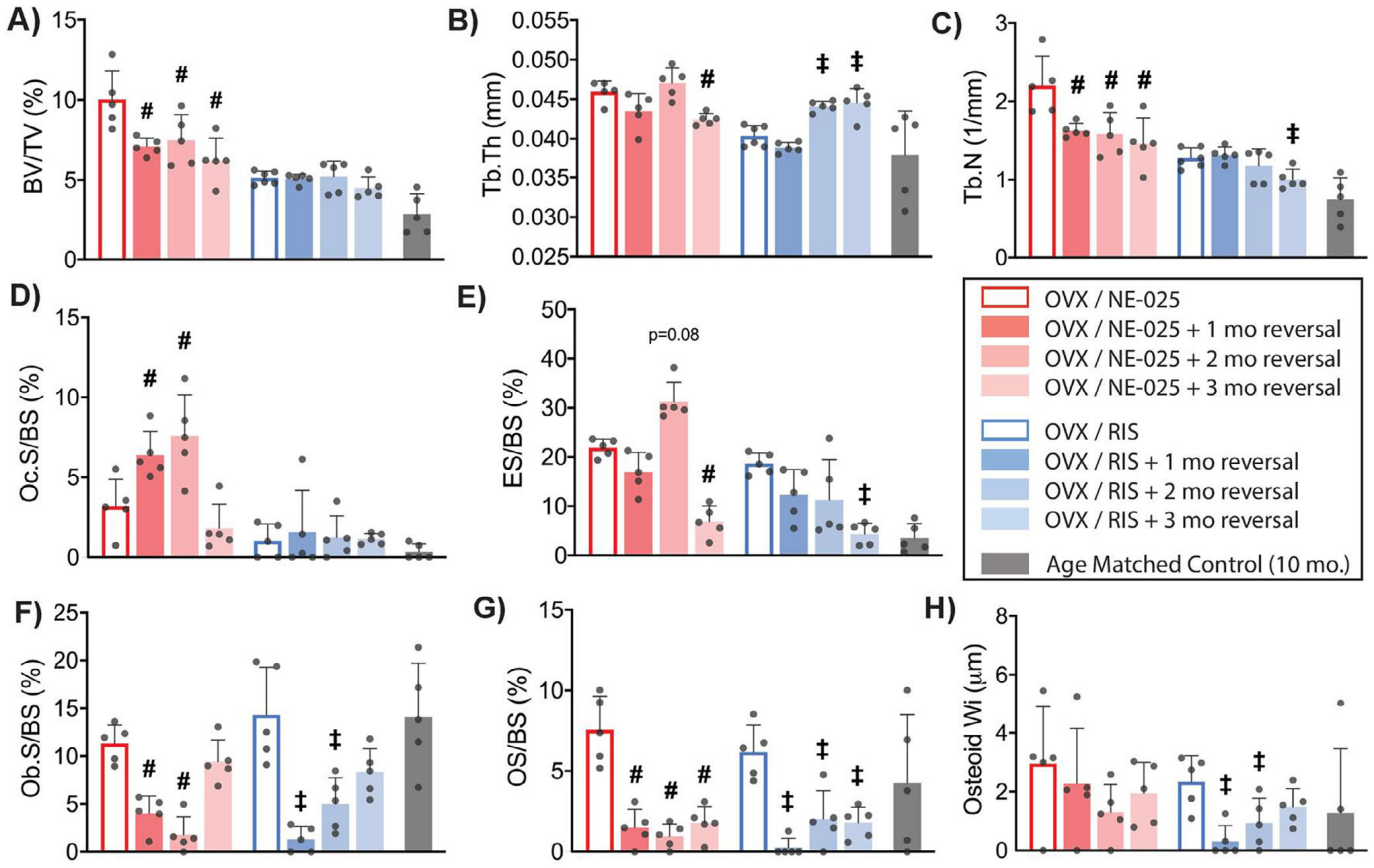
Long‐term changes in cancellous bone after stopping bisphosphonate treatment in OVX mice; data shown for 1, 2, and 3 months after stopping NE‐58025 (NE‐025) or RIS treatment. Graphs *A*‐*C* show changes in trabecular bone architecture from μCT analyses and graphs *D*‐*H* show changes in osteoclast and osteoblast indices. Comparisons are versus the 3‐month OVX/treatment‐starting points. Data shown as mean ± SD. (*A*) Cessation of NE‐025 treatment led to rapid onset of trabecular bone loss with a significant reduction in BV/TV starting within 1 month. (#*p* = 0.03 for OVX/NE‐025 + 1‐month reversal; *p* = 0.04 for OVX/NE‐025 + 2‐month reversal and *p* = 0.003 for OVX/NE‐025 + 3‐month reversal). Stopping RIS treatment did not result in bone loss. (*B*,*C*) Bone loss was principally caused by a reduction in Tb.N. (*D*) Stopping NE‐58025 treatment resulted in a 200% increase in Oc.S/BS within 1 month and a 250% increase by 2 months (#*p* = 0.04 for OVX/NE‐025 + 1‐month reversal and *p* = 0.02 for OVX/NE‐025 + 2‐month reversal), and (*E*) ES/BS increased within 2 months of stopping treatment (*p* = 0.08). (*F*) Osteoblast surface was significantly decreased after 1 and 2 months of NE‐58025 reversal (#*p* = 0.009 for OVX/NE‐025 + 1‐month reversal and *p* = 0.001 for OVX/NE‐025 + 2‐month reversal). Ob.S/BS also decreased after 1 and 2 months of reversal for RIS groups (‡*p* = 0.0002 for OVX/RIS + 1‐month reversal and *p* = 0.02 for OVX/RIS + 2‐month reversal). (*G*) OS/BS declined for all time points of reversal for NE‐58025 (#*p* = 0.008 for OVX/NE‐025 + 1‐month reversal, *p* = 0.001 for OVX/NE‐025 + 2 ‐month reversal, and *p* = 0.03 for OVX/NE‐025 + 3‐month reversal) and RIS (‡ *p* = 0.0002 for OVX/RIS + 1‐month reversal, *p* = 0.03 for OVX/RIS + 2‐month reversal, and OVX/RIS + 3‐month reversal). (*H*) Osteoid Wi decreased at 1 and 2 months of reversal (‡*p* = 0.002 for OVX/RIS + 1‐month reversal and *p* = 0.04 for OVX/RIS + 2‐month reversal). BV/TV = trabecular bone volume fraction; ES/BS = eroded surface/bone surface; Ob.S/BS = osteoblast surface/bone surface; Oc.S/BS = osteoclast surface/bone surface; OS/BS = osteoid surface/bone surface; Osteoid Wi = osteoid width; OVX = ovariectomy; RIS = risedronate; Tb.Th = trabecular thickness; Tb.N = trabecular number.

In these reversal studies, all bone properties observed after withdrawal of NE‐58025 or RIS are expressed relative to the bone parameters at the end of the 3‐month‐treatment period (i.e., time zero for reversal). Cessation of NE‐58025 treatment in OVX mice led to the rapid onset of resorption activity and trabecular bone loss. Bone loss occurred quickly after stopping NE‐58025 treatment with BV/TV, and Tb.N decreased by approximately 35% at 1 month posttreatment (*p* = 0.03; Fig. 5*A*‐*C*); bone architecture then remained stable for the duration of the study period. Osteoclast surface and eroded surface were increased markedly at 1 month and 2 months posttreatment in NE‐58025 mice, respectively (Fig. 5*D*,*E*; *p* = 0.04 and *p* = 0.02). At 3 months after stopping NE‐58025 treatment, both osteoclast surface and eroded surface levels were similar to age‐matched controls. In contrast, stopping RIS treatment did not lead to bone loss or any increase of osteoclast or eroded surfaces during the 3‐month posttreatment period (Fig. 5*A*‐*E*).

#### Cortical bone

Withdrawal of either BP did not lead to significant marrow cavity expansion/diaphyseal cortical bone loss or increased eroded surface (Tables 4B and 5B). Accordingly, no changes in diaphyseal mechanical properties were found among bones in the reversal study (Table 6B).

## Discussion

In the current study, we found that NE‐58025, a BP with a low HAP binding affinity and moderate potency for osteoclast inhibition, successfully suppressed OVX‐induced bone loss. Surprisingly, NE‐58025 prevented bone loss more effectively than RIS in this study. Once treatment was stopped, the antiresorptive effects of NE‐58025 reversed very rapidly with increases in osteoclastic activity and bone loss beginning 1 month after ceasing NE‐58025 treatment. This contrasts markedly with the situation after withdrawing RIS treatment, where neither bone resorption nor did bone loss resume after stopping treatment during the 3‐month course of the reversal study.

Current clinical BPs owe their exceptional efficacy as antiresorptive agents to two major properties: (i) ability to inhibit osteoclastic activity by binding and blocking FPPS, which interferes with osteoclast resorption of bone, and (ii) moderate‐to‐high affinity to bone mineral, which allows them to selectively target and adhere strongly to bone surfaces in particular at sites of highest bone turnover. This HAP binding affinity results in very long functional half‐lives for suppression of bone resorption. Reactivation times for bone resorption after stopping BP treatment are reported to be several months or longer in rodents^(^ ^52^ ^)^ and years in humans.^(^ ^22^, ^23^, ^26^, ^28^, ^53^ ^)^ BPs with low HAP affinity (thus rapid biological clearance) and potent osteoclast inhibition have not been developed for clinical use because of their short functional half‐lives. However, they may offer potential advantages with regard to reversibility.^(^ ^33^ ^)^ The agent we tested in the current study, NE‐58025, is one such compound. The FPPS IC_50_ for NE‐58025, the concentration needed for 50% inhibition of FPPS in biochemical assays, has been reported at as 42nM, which is a relatively high potency in the same general range as zoledronate and RIS (4.1 and 5.7nM, respectively).^(^ ^48^, ^67^ ^)^ Whereas, alendronate has a much lower FPPS inhibition (260nM), but a very high affinity for bone mineral (second only to zoledronate), which allows it to function as an effective antiresorptive.^(^ ^47^ ^)^ RIS has the lowest bone affinity among current clinical BPs.^(^ ^36^, ^51^ ^)^

Our test compound, NE‐58025, has a different combination of functional attributes – much lower HAP affinity than any of the current clinical BPs and high FPPS inhibition.^(^ ^33^, ^48^ ^)^ This allowed us to preform proof‐of‐concept in vivo tests for a reversible BP with low HAP binding affinity and high FPPS inhibition. The affinity of a BP for bone mineral is typically modulated by the R_1_ group (i.e., a hydroxyl group for high‐affinity BPs) attached to the P‐C‐P backbone of the BP to allow for stronger or weaker binding to the bone.^(^ ^33^, ^36^ ^)^ In the case of NE‐58025, the R_1_ substituent is incorporated in a cycloalkyl ring structure that hinders the binding of the phosphonate groups to calcium on the bone mineral surface.^(^ ^33^, ^49^, ^67^ ^)^ The R_2_ group of NE‐58025 is analogous to that of RIS in that it orients a basic nitrogen moiety similarly for binding to FPPS enzyme, hence its comparable FPPS inhibition activity and antiresorptive capabilities.^(^ ^48^ ^)^ The benefit of the low HAP binding of NE‐58025 is the potential for rapid dissolution from bone. The challenge with such a compound is that it required daily dosing at a high concentration in these studies to prevent OVX‐induced bone loss; dosing 3 times/week did not significantly reduce OVX‐induced bone loss.

In the current studies, RIS treatment substantially reduced bone loss long‐term after estrogen withdrawal (~35% reduction from baseline BV/TV), as expected. This level of bone loss attenuation is comparable with that reported in numerous previous studies using RIS after OVX in rats and mice, even when RIS doses were much higher (up to 10 times) than those used in the current study.^(^ ^68^, ^69^ ^)^ In contrast, NE‐58025 at the dose tested completely stopped bone loss after OVX in our studies. The reasons for this difference in vivo are not yet completely understood. However, differences in HAP binding affinity and local bioavailability may be key.^(^ ^33^ ^)^ McClung and Ebetino^(^ ^48^ ^)^ reported that relative affinity of NE‐58025 for bone mineral is substantially lower than RIS (7 vs 10 min, respectively, measured by retention time on an HAP column). Lawson et al^(^ ^33^ ^)^ report even greater HAP affinity for RIS (~16 min). In other words, RIS adheres more strongly to the mineral on bone surfaces and should stay more fixed to bone than NE‐58025, which would more readily desorb from bone surfaces. Thus, it seems reasonable to speculate that there is a constant amount of NE‐58025 releasing from the bone surfaces that can be presented to nearby osteoclasts consistently, which would provide more effective, local dosing at resorbing sites over time. Further studies are needed to test this idea.

Plotkin, Bellido, and colleagues have shown that BPs can prevent osteocyte apoptosis induced by glucocorticoids^(^ ^38^, ^70^ ^)^ and unloading^(^ ^71^ ^)^, and Follet et al^(^ ^40^ ^)^ have shown that BP acutely attenuated osteocyte apoptosis in fatigue‐loaded bone. Osteocyte apoptosis has been shown to be a key trigger for the activation of new resorption in response to bone microdamage or estrogen loss, and inhibition of this apoptosis prevents recruitment and activation of new osteoclasts.^(^ ^54^, ^72^, ^73^, ^74^, ^75^, ^76^ ^)^ However, we think it is unlikely that the attenuation of osteocyte apoptosis by BPs is implicated in the long‐term antiresorptive actions of either RIS or NE‐58025 in this study. Follet and colleagues^(^ ^40^ ^)^ reported that the BP antiapoptotic effect on osteocytes in their bone fatigue studies was not sustained for long because osteocyte death increased to nontreated control levels within a week despite continued BP treatment. Thus, the antiapoptotic effects of BPs on osteocytes appears to be of short‐duration and transient, lasting from days to weeks after a challenge is introduced to the bone. The current long‐term resorption prevention studies lasted for 3 months after OVX, which is expected to be well after the acute effects of OVX on triggering osteocyte apoptosis. Therefore, we assume likewise for potential antiapoptotic effects of either RIS or NE‐58025 and did not examine osteocyte apoptosis in these tissues.

Impaired bone quality (i.e., material properties) after long‐term suppression of bone remodeling with BP has been shown in animal models and in tissues from patients treated long‐term with BPs.^(^ ^10^, ^12^, ^14^, ^16^, ^77^, ^78^ ^)^ This impairment is hypothesized to drive the development of AFFs for a small proportion of patients receiving long‐term BP treatment.^(^ ^12^, ^14^, ^16^ ^)^ In their seminal studies, Mashiba and colleagues^(^ ^79^ ^)^ found that long‐term suppression of bone remodeling with alendronate or RIS treatment in healthy young adult dogs allowed fatigue microdamage to accumulate in bone with normal daily activities. Bone microdamage accumulation decreases fracture resistance of the bone material.^(^ ^80^ ^)^ Suppressed bone turnover also results in increases of bone matrix mineralization and loss of tissue heterogeneity, which will also decrease material fracture resistance.^(^ ^14^ ^)^ In a recent, remarkable study, Donnelly and coworkers^(^ ^16^ ^)^ were able to compare compositional and material properties of femoral bone samples acquired from BP‐treated patients with AFFs to those from patients with typical osteoporotic fractures both with and without BP treatment. They found that cortical bone tissue from BP‐treated women with AFF was more highly mineralized than that from BP‐treated women with typical osteoporotic fractures. Furthermore, fracture mechanics studies showed that tissue from patients treated with BPs had significantly reduced fracture toughness, and the osteonal boundaries were far less effective at limiting crack growth than bone from BP‐naïve subjects.

A potential solution that has been widely discussed in the clinical literature to mitigate the adverse biomechanical effects of prolonged suppression of bone remodeling is a so‐called drug holiday. The concept is that a temporary discontinuation of BP treatment will allow bone turnover to restart and thus remodel and replace foci of bone that have sustained microdamage or where material properties have become impaired. Whether this would actually occur in situ remains unknown. The data to date regarding the efficacy and usefulness of a BP drug holiday are confusing and often contradictory. Dennison and colleagues^(^ ^81^ ^)^ recently reported that the risk of new fractures could be as much as 40% higher in subjects who stopped BP treatment caused by resumed bone loss once long‐term suppressed resorption is reactivated. However, Black and colleagues^(^ ^22^, ^23^ ^)^ found no difference in nonvertebral osteoporotic fractures when comparing subjects who continued long‐term alendronate treatment with others who stopped alendronate after an initial 5‐year‐treatment period, although they did note an increased risk for clinical vertebral fractures among discontinuers. In a recent large population cohort study, Adams and colleagues^(^ ^82^ ^)^ found that women who took a holiday from BP treatment after 3 or more years did not appear to be at increased risk of osteoporosis‐related fragility fracture, hip, or vertebral fractures compared with ongoing BP users.

Studies reviewing the effects of a BP drug holiday on the incidence of AFF have more consistent results. In their large cohort study, Adams et al^(^ ^82^ ^)^ found that the incidence of AFF was significantly higher in BP users (mostly treated with alendronate), who continued versus discontinued use of the drug, suggesting a strong benefit of a drug holiday. Similarly, Anagnostis et al^(^ ^83^ ^)^ examined AFF cases and controls (i.e., conventional subtrochanteric or other osteoporotic fractures) and found that after BP withdrawal the risk of AFF was reduced by 72% per year after last use of the drug. Such results are intriguing and could imply that renewed bone remodeling may indeed be beneficial in repairing the bone material defects that result from long‐term remodeling suppression. However, the fundamental question of whether bone quality can be restored by a BP drug holiday remains unanswered and awaits future study.

Although the data regarding the issue of discontinuing BPs remain contradictory, recent clinical and animal studies show that stopping the antiresorptive denosumab results in an increase in bone resorption markers, and in some cases resumed bone loss to levels that surpass pretreatment with rebound vertebral fractures observed in some postmenopausal woman after discontinuation of denosumab.^(^ ^84^, ^85^, ^86^, ^87^ ^)^ In the current studies, NE‐58025 discontinuation resulted in a rapid increase in osteoclast surface and eroded surface, followed by a decrease in BV/TV to values slightly lower than baseline controls after 3 months of stopping treatment. However, resorption indices at this time were reduced to age‐matched control level, suggesting that NE‐58025 reversal might reach a steady state within a few months after stopping treatment.

A BP that is both effective at preventing bone loss and rapidly reversible, such as NE‐58025, may have clinical use as an antiresorptive agent with an on/off profile of weeks rather than months to years—time frames typical of currently used BPs, including alendronate and RIS. In addition to use in osteoporosis, lower affinity antiresorptive agents may also be useful in the treatment of other bone diseases with high levels of bone resorption, such as rheumatoid arthritis and oncology, including the potential prevention of various cancers.^(^ ^3^, ^88^ ^)^ Our studies showed that NE‐58025 needed to be administered daily to completely prevent bone loss. This seems unlikely to be well tolerated by patients. However, there are other methods (i.e., nanoparticle systems or sustained release capsules) that could be explored to increase the half‐lives of these drugs and find the best functional compromise between sustained action and rapid reversibility. These studies are the first to our knowledge to show the antiresorptive capabilities and rapid reversibility of a low HAP affinity BP. Based on these findings, it is reasonable to conclude that low HAP affinity/high FPPS potency BPs might be further explored for clinical use. The rapid reversal of antiresorptive effects after stopping treatment with NE‐58025 may prove to be advantageous by providing insight into whether a drug holiday can actually improve bone quality and testing whether reactivated remodeling can in fact restore bone material properties and ultimately decrease the risk of AFF. This group of BPs may prove valuable for exploring the biomechanical and compositional effects of a drug holiday, as well as titrating the duration of a drug holiday. Such insights could have the potential to alter clinical paradigms for treating osteoporosis in patients and decrease the incidence of AFF.

## AUTHOR CONTRIBUTIONS

**Abigail Coffman:** Conceptualization; data curation; formal analysis; investigation; methodology; project administration; software; validation; visualization; writing‐original draft; writing‐review & editing. **Jelena Basta‐Pljakic:** Data curation; formal analysis; software; validation. **Rosa Guerra:** Data curation; formal analysis. **Frank Ebetino:** Methodology; resources; writing‐review & editing. **Mark Lundy:** Methodology; resources; writing‐review & editing. **Robert Majeska:** Conceptualization; methodology; supervision; writing‐review & editing. **Mitchell Schaffler:** Conceptualization; funding acquisition; methodology; project administration; supervision; validation; visualization; writing‐original draft.

## Conflict of Interest

Frank H Ebetino and Mark W Lundy are employees and stockholders of BioVinc, LLC. BioVinc does not hold a financial or licensing interest in the NE‐58025 bisphosphonate used in this research.

## Supporting information


**Supplementary Table S1** Changes in cancellous bone volume and bone cell surface measurements for short‐term NE‐58025 studies to determine effective dose for preventing OVX‐induced bone loss. Sham data shows that NE‐58025 has no effect on bone. Data shown as mean ± SD. * *p* = 0.001 for OVX/PBS for BV/TV. *p* = 0.002 for OVX/PBS and *p* = 0.001 for OVX/200 μg/kg, 7d/wk and Sham/PBS for Tb.Th. *p* = 0.001 for OVX/PBS for Tb.N.
