# Supplementary material for: A Bisphosphonate With a Low Hydroxyapatite Binding Affinity Prevents Bone Loss in Mice After Ovariectomy and Reverses Rapidly With Treatment Cessation
Source: JBMR Plus. 2021 Mar 3;5(4):e10476. doi: 10.1002/jbm4.10476 (PMC8046044; doi:10.1002/jbm4.10476)
Supplement: Supplementary file 1 — Supplementary Table S1 Changes in cancellous bone volume and bone cell surface measurements for short‐term NE‐58025 studies to determine effective dose for preventing OVX‐induced bone loss. Sham data shows that NE‐58025 has no effect on bone. Data shown as mean ± SD. * p = 0.001 for OVX/PBS for BV/TV. p = 0.002 for OVX/PBS and p = 0.001 for OVX/200 μg/kg, 7d/wk and Sham/PBS for Tb.Th. p = 0.001 for OVX/PBS for Tb.N. [file JBM4-5-e10476-s001.docx]

**Supplemental Table.** Changes in cancellous bone volume and bone cell surface measurements for short-term NE-58025 studies to determine effective dose for preventing OVX-induced bone loss. Sham data shows that NE-58025 has no effect on bone. Data shown as mean ± SD. ^*^ p=0.001 for OVX/PBS for BV/TV. p=0.002 for OVX/PBS and p= 0.001 for OVX/200 μg/kg, 7d/wk and Sham/PBS for Tb.Th. p = 0.001 for OVX/PBS for Tb.N.

| Parameter | Baseline | OVX/PBS | Sham/PBS | OVX/NE-025 200 μg/kg, 7d/wk | Sham/NE-025 200 μg/kg, 7d/wk |
| --- | --- | --- | --- | --- | --- |
| BV/TV,% | 8.42 ± 2.2 | 4.5 ± 0.7 ^*^ | 6.0 ± 0.8 | 6.5 ± 0.8 | 8.5 ± 1.2 |
| Tb. Th, mm | 0.046 ± 0.003 | 0.041 ± 0.002 ^*^ | 0.038 ± 0.002 ^*^ | 0.039 ± 0.002 ^*^ | 0.043 ± 0.002 |
| Tb. N, 1/mm | 1.8 ± 0.4 | 1.1 ± 0.2 ^*^ | 1.6 ± 0.1 | 1.6 ± 0.2 | 2.0 ± 0.2 |
